# Longitudinal analysis of resting energy expenditure and body mass composition in physically active children and adolescents

**DOI:** 10.1186/s12887-022-03326-x

**Published:** 2022-05-10

**Authors:** Edyta Łuszczki, Anna Bartosiewicz, Maciej Kuchciak, Katarzyna Dereń, Łukasz Oleksy, Olga Adamska, Artur Mazur

**Affiliations:** 1 Institute of Health Sciences, Medical College of Rzeszow University, Rzeszow, Poland; 2 Institute of Physical Culture Sciences, Medical College of Rzeszow University, Rzeszow, Poland; 3 Physiotherapy and Sports Centre, Rzeszow University of Technology, Rzeszów, Poland; 4 Department of Orthopaedics and Rehabilitation, Medical University of Warsaw, Warsaw, Poland; 5 Institute of Medical Sciences, Medical College of Rzeszow University, Rzeszow, Poland

**Keywords:** Children and adolescents, Indirect calorimetry, Metabolism, Physical activity, Resting energy expenditure

## Abstract

**Background:**

Monitoring body composition and changes in energy expenditure during maturation and growth is significant, as many components can influence body structure in adulthood. In the case of young players, when these changes can influence their strength and power, it seems to be equally important. Our aim was to examine whether resting energy expenditure (REE) and body composition would change after 10 months from baseline in physically active children and adolescents.

**Methods:**

We obtained data from 80 children and adolescents aged 9 to 17 years at two measurement points: the baseline in September 2018 and after 10 months in July 2019. The study was carried out using a calorimeter (Fitmate MED, Cosmed, Rome, Italy), a device used to assess body composition using by the electrical bioimpedance method by means of a segment analyzer (TANITA MC-980). The Student’s t-test and linear regression analysis were used. Using the stepwise forward regression procedure, the selection of factors in a statistically significant way that describes the level of REE was made.

**Results:**

We noticed that REE was not significantly different between baseline (1596.94 ± 273.01 kcal) and after 10 months (1625.38 ± 253.26 kcal). When analyzing the difference in REE between studies girls, we found body height as a significant predictor. The results of our study show a negative relationship between growth and REE. Differences between sexes and age in REE between baseline and after 10 months were not significant.

**Conclusions:**

Our study involving physically active children and adolescents, which used repeated objective measures and longitudinal statistical modeling to analyze them, was unable to demonstrate any interaction between body weight change, body composition measurements, and REE.

**Supplementary Information:**

The online version contains supplementary material available at 10.1186/s12887-022-03326-x.

## Background

According to experts from the European Food Safety Authority (EFSA), Food and Agriculture Organization of the United Nations (FAO), World Health Organization (WHO), United Nations University (UNU) and the Institute of Medicine in the United States, the basis for determining energy demand is total energy expenditure (TEE) including all its components, i.e. energy expenditure related to resting energy expenditure (REE), physical activity, the thermal effect of food as well as tissue building and synthesis [1]. Furthermore, the energy expenditure associated with body development and intensive physical activity also increases demand. The measurement of REE is usually the first step in determining the energy demand for people training in various sports disciplines. REE becomes a significant contribution to total energy expenditure even for active people, such as those training for endurance sports (marathon, swimming, rowing, etc.). For most people, REE accounts for almost 60 to 70% of total energy demand [2]. Therefore, the definition of REE can serve as a valuable tool in the development of food rations or nutrition plans to improve athletic performance and prevent body weight loss in physically active children and adolescents.

A very widespread method of determining REE is indirect calorimetry, based on the principle of the body using the energy obtained from the oxidation of nutrients, which is associated with the consumption of oxygen and the release of carbon dioxide in quantities proportional to the expenditure of energy, and therefore the relationship between the rate of oxygen absorption of the body and the amount of energy released in the oxidation processes [3, 4].

Physical activity (PA) refers to any type of musculoskeletal activity which raises energy expenditure above basal values. It is considered to be the component that shows the greatest variability relative to total energy expenditure (TEE). Monitoring body composition and changes in energy expenditure during maturation is significant, as many components can influence body structures in adulthood. Furthermore, it is important for young football players that these changes can influence their strength and power [5]. The strong relationship between REE and muscle mass has been the subject of research by many authors. The literature shows fat-free mass (FFM) as the strongest indicator affecting REE [6–9]. It has been shown that FFM can have a strong impact on energy requirements. Age is also a parameter that has been confirmed in numerous studies to influence REE [10, 11]. This may be associated with pubertal spurt and a greater amount of FFM in this group in older children and adolescents who train longer and thus have more muscle fibers. The literature also indicates the effect of puberty on REE [12]. Research in soccer players indicates that REE increases by approximately 400 kcal/day from the chronological ages of 10 to 13 [13].

Indirect calorimetry has been implemented by Japanese researchers to precisely estimate the REE in children and adolescents. The study was carried out among 221 children aged 6 to 17. The researchers applied multiple regression analysis using a combination of age, sex, body weight, and body height, or a combination of age, sex, lean mass, and fat mass. The research showed that REE of Japanese children increased with age, both in boys and girls, and there was a significant gender difference in the age group 12 to 17 [14]. Furthermore, Broadney et al. showed that the differences in REE of the children studied result from differences in body composition [15]. They demonstrated the age dependence of REE in Caucasian American children. There are very few reports in the literature regarding the determination of REE in the same group in two different time moments using indirect calorimetry in a population of children and adolescents who play football.

Our study is one of the few conducted in Poland using indirect calorimetry to calculate REE in physically active children and adolescents. The determination of the resting energy expenditure in a group of children and adolescents, especially those who play sports, is the basis for a precise determination of the energy demand on food intake, which is directly related to the health and physical condition of the respondents [4]. When evaluating REE of children and adolescents, factors that can influence REE should be considered to ensure appropriate interpretation. Age, sex, body size, and body composition, specifically fat-free mass (FFM), have been identified as the most significant factors, with the population group and PA as possible contributing factors [16, 17]. However, there is little information on the effect of long-term weight gain and growth on REE in children and adolescents, and only a few studies have tracked longitudinal changes in REE during childhood, particularly during puberty. Previous studies have been limited by their cross-sectional design [11, 18] or small sample size [19]. After REE, PA is the second largest and most variable component that contributes to total energy expenditure. It refers to any voluntary and involuntary bodily movements produced by muscle contraction [20]. Apart from the direct effect of PA on total energy expenditure, evidence exists [21, 22] that PA may influence REE, and its effects can last for hours or days (referred to as the excess post-exercise O2 consumption [EPOC]) [20, 22, 23].

Therefore, the purpose of our study was a longitudinal analysis of resting energy expenditure and body composition in physically active children and adolescents. We hypothesized that: 1) the REE would increase during the follow-up period; 2) there would be a difference in body composition and change in body weight over time between the sexes.

## Methods

### Participants

A study was carried out in the 2018/2019 school year in a randomly selected (from 7 sports schools in this region) sports school in Rzeszów (Poland) and involved healthy children and adolescents aged 9 to 17 years. All data were obtained at two moments: baseline (T0)– September 2018 and after 10 months (T1) – June, July 2019. The inclusion criteria were age 9–18 years, training football about three times a day and playing a game once a week, and consent from the parent or guardian to participate in the study.

The study group consisted of 80 students (17 girls and 63 boys) aged 9 to 17. The study methodology has been published in detail [24].

Study participants and their legal guardians received verbal and written information on the objectives, risks, and benefits of the study. Both guardians and participants gave their informed written consent to participate in the study.

### Assessments

Research was carried out at the Laboratory for Innovative Research in Dietetics (Centre for Innovative Research in Medical and Natural Sciences, University of Rzeszow, Rzeszow, Poland). Body weight, body height, and REE assessments of the study sample have been published elsewhere [24]. Body height was measured 3 times with an accuracy of 0.1 cm (by a portable Seca 213 stadiometer). REE was measured by the indirect calorimetry method using an indirect calorimeter (Fitmate MED, Cosmed, Rome, Italy). The Fitmate Med device was validated and showed a very high reliability of the measurements obtained [25]. The results obtained using Fitmate Med are comparable to those obtained with the Douglas bag system, which uses a sensor to measure VCO2 [26]. A study by Campbell et al. examined the validity and reliability of the Fitmate device. On the first day, two 15 minute tests were performed, then on the second day (within a week after performing 1 test) another test was carried out. To assess the reliability of the test, intraclass correlation coefficients (ICC) and standard error measurement (SEM) were used, while Anova analyzed systematic error. Relative consistency was accepted with the SEM and ICC values (0.981 and 0.946, responding during the day and between). Moreover, no systematic error was found between the measurements [25]. In order to properly use the device in the pediatric population, a request for guidance was sent to the manufacturer. According to the instructions, it was recommended to use disposable antibacterial filters with rubber mouthpieces to improve mouth grip and limit the risk of air leakage by using a reusable mask (a petite/pediatric size).

All recommendations concerning preparations for the study were outlined during the meeting with participants and parents / guardians, including rest, refraining from eating meals 12 hours before the test, refraining from drinking beverages with caffeine content for the last 48 hours before the test, as well as refraining from participating in physical activity for the previous 12 hours.

### Body composition and body mass index

Body composition was measured using the electrical bioimpedance method (6.25 kHz, 50 kHz, 90 μA) using a calibrated segment analyzer (Tanita MC-980 PLUS MA, Tokyo, Japan) with an accuracy of 0.1 kg/ 0.1%. Tanita MC 980 has approvals for medical use and meets the NAWI and CLASS III standards and the MDD 93/42/EEC directive, as well as the CE0122 EU certificate [27]. The results obtained using the Tanita Analyzer for studies involving children are consistent with those obtained from Dual Energy X-ray Absorptiometry (DXA) [13, 28–30]. The analyzer is equipped with 8 electrodes, 4 of which are built into the platform, while the others are in holders. Participants were asked to remove their footwear and socks, then the skin on their feet was cleaned so that the measurement was carried out correctly. All test participants were in their underwear, stood still on the platform, in the designated places. According to the Tanita MC980 PLUS MA manual, the machine was set as vertically as possible to ensure accurate measurement. The device was set and adjusted so that the level indicator was in the center of the level meter. Participants stood on the platform barefoot, upright, with straight legs, placing their feet so that they touched the front and rear electrodes, making sure that the weight of the body was evenly distributed between both feet. In their hands, the examined person held handles positioned away from the body at an angle of 35 °-40 °. A person’s measurement is taken while in a standing position with the elecrodes in contact with bare feet and hands. The device automatically measures body weight and then impedance. The Commuter software (a microprocessor) imbedded in the product uses the measured impedance, the participant’s sex, body height, fitness, age, and the weight to determine body fat percentage based on equation formulas. The Tanita reference method is DXA. Through multiple regression analysis, Tanta has derived standard formulas to determine the percentage of body fat. The Tanita equations are generalized for standard adults, athletes, and children.

Body mass index (BMI) was calculated as body weight (kg)/ height (m)2. The definitions of body mass deficiency, normal body weight, overweight, and obesity were based on the recommendations of the Centers for Disease Control and Prevention [31].

### Arterial blood pressure

Blood pressure was measured three times according to the recommendations of the National High Blood Pressure Education Program Working Group in Children and Adolescents (NHBPEP) [32], using a Welch Allyn 4200B-E2 blood pressure meter (Aston Abbotts, UK) with cuffs sized to fit the shoulders of the participants. The average of three measurements was calculated for each person tested.

### Statistical analysis

The results of the study were obtained using descriptive statistics: number (n), average, Me - median and standard deviation (SD). Both parametric and non-parametric tests were used to analyze the variables. The choice of the parametric test depended on fulfilling its basic assumptions, i.e., the conformity of the tested variable with normal distribution, which was verified by the Kolmogorov–Smirnov test. The Student’s t-test was used for normally distributed variables. In addition, linear regression analysis was used. Using the stepwise forward regression procedure, the selection of factors in a statistically significant way that describes the level of REE was made. Statistical significance was established as a *p* value less than 0.05. Calculations were performed with the Statistica 10.0 tool (StatSoft, Inc.Tulsa, Oklahoma, United States).

### Causal framework

The multivariate models were adjusted for a set of a priori–determined covariates that included age, body height, BMI, fat mass, FFM, total body water, hip circumference, waist circumference, systolic pressure, and diastolic pressure.

Because age and sex are a strong determinant of REE, we controlled for age and sex in a basic model. Potential confounders were first selected based on previous studies, as well as a literature search. Confounding variables were included in the final model if the covariate was associated with exposure at *p* < 0.05 or a priori (if there was a strong theoretical or clinical reason to keep them in the model). As there were too many variables, a stepwise procedure was employed to include potential confounding variables that have a detectable effect on the association of interest while retaining the above-mentioned variables in the model. We also performed a formal sensitivity analysis, as described by Lin et al., to assess the potential effect of unmeasured confounding on our results. It is also possible that residual confounders remained after inaccurate measurement of physical activity, smoking status, or blood pressure.

## Results

### Characteristics of the study group

A total of 80 respondents aged 9 to 17 years were surveyed twice, 21.3% of whom were girls (*N* = 17), and 78.7% were boys (*N* = 63). The mean age of the respondents at baseline was 12.04 ± 2.26 years and after 10 months - 12.32 ± 2.32 years. The mean age of the girls at baseline and after 10 months was significantly higher (*p* < 0.05) than the mean age of the boys (Table 1).

**Table 1:** Characteristics of the study group by age and sex in T0 and T1

	Sex	N	Average	SD	SE	t	df	***p***
**Age** Baseline	Girls	17	14.12	1.65	0.40	4.852	78	< 0.0001*
	Boys	63	11.48	2.07	0.26			
**Age** After 10 months	Girls	17	14.53	1.81	0.44	4.947	78	< 0.0001*
	Boys	63	11.78	2.09	0.26			

*SD* standard deviation, *SE* standard error

^*^indicate significant values (*p* < 0.05)

### The findings

In Tables 2 and 3 the differences between the groups are presented.

**Table 2:** Between-group differences from T0 to T1 by age

Variables	T0							T1							***p***
	Average	SD	Min.	Max	Q1	Q2 (Me)	Q3	Average	SD	Min.	Max	Q1	Q2 (Me)	Q3	
**Total (*N*** **= 80)**															
**REE [kcal]**	1596.94	273.01	1187.00	2639.00	1415.00	1549.00	1734.25	1625.38	253.26	1129.00	2682.00	1453.50	1597.00	1745.00	0.1520
**Body height [cm]**	153.70	13.19	127.00	184.00	143.00	154.00	163.00	155.50	12.56	129.00	186.00	145.25	155.50	164.75	< 0.0001*
**Body weight [kg]**	44.15	11.69	24.60	79.70	34.70	43.60	52.30	46.15	11.96	25.30	82.70	36.50	45.80	54.63	< 0.0001*
**BMI [kg/m2]**	18.31	2.42	13.60	24.90	16.70	18.00	19.45	18.73	2.44	14.50	25.50	16.93	18.35	20.15	< 0.0001*
**FM [%]**	19.80	4.72	12.90	34.50	16.55	19.10	22.78	20.22	4.54	13.50	33.90	16.83	19.30	22.45	0.0597
**FFM [kg]**	35.24	9.07	19.60	61.20	28.70	34.40	39.90	36.64	9.14	20.30	63.50	30.20	36.10	41.15	< 0.0001*
**TBW [kg]**	25.80	6.64	14.30	44.80	21.00	25.20	29.20	26.82	6.69	14.90	46.50	22.10	26.45	30.15	< 0.0001*
**HC [cm]**	78.49	9.04	64.00	97.00	71.00	78.00	85.00	81.16	9.28	66.00	108.00	74.00	79.00	89.00	< 0.0001*
**WC [cm]**	61.45	7.56	21.00	74.00	57.00	61.00	66.00	62.87	9.24	6.00	84.00	59.00	62.00	67.00	0.1520
**SBP [mmHg]**	108.03	13.13	85.00	141.00	99.00	106.50	114.00	109.90	11.92	87.00	146.00	101.00	110.00	118.00	0.1597
**DBP [mmHg]**	62.66	6.84	47.00	78.00	57.00	62.00	68.75	63.30	7.04	50.00	82.00	58.00	64.00	67.00	0.4540
**AGE = 9–12 years (*N*** **= 50)**															
**REE [kcal]**	1518.86	193.22	1187.00	2054.00	1363.75	1500.50	1629.75	1536.56	187.84	1129.00	2083.00	1407.50	1536.50	1625.75	0.4117
**Body height [cm]**	145.84	8.48	127.00	169.00	139.00	145.00	153.25	148.22	8.57	129.00	170.00	141.75	146.00	155.00	< 0.0001*
**Body weight [kg]**	37.51	7.60	24.60	56.90	31.48	36.00	41.60	39.31	7.86	25.30	59.50	32.83	37.75	45.30	< 0.0001*
**BMI [kg/m2]**	17.43	2.22	13.60	23.80	15.98	17.00	18.70	17.73	2.11	14.50	23.70	16.33	17.35	18.93	< 0.0001*
**FM [%]**	19.26	3.85	13.60	33.40	16.78	18.55	20.53	19.31	3.46	13.50	32.70	16.90	18.90	20.85	0.7693
**FFM [kg]**	30.12	5.25	19.60	42.30	25.68	29.65	33.95	31.58	5.57	20.30	45.10	26.53	31.40	35.68	< 0.0001*
**TBW [kg]**	22.05	3.85	14.30	31.00	18.78	21.70	24.85	23.12	4.07	14.90	33.00	19.45	23.00	26.15	< 0.0001*
**HC [cm]**	73.18	5.89	64.00	86.00	69.75	72.00	78.00	75.98	6.36	66.00	94.00	71.75	75.00	79.00	< 0.0001*
**WC [cm]**	58.22	7.44	21.00	73.00	55.00	58.00	61.25	60.12	9.75	6.00	81.00	57.00	59.00	63.25	0.2132
**SBP [mmHg]**	105.62	12.34	85.00	141.00	95.75	104.00	114.00	105.56	10.90	87.00	132.00	98.00	104.00	111.50	0.9661
**DBP [mmHg]**	60.46	6.49	47.00	74.00	56.00	59.00	65.00	61.46	6.63	50.00	80.00	56.00	61.50	66.00	0.4001
**AGE = 13–17 years (*N*** **= 30)**															
**REE [kcal]**	1727.07	334.56	1219.00	2639.00	1540.25	1673.00	1870.25	1773.40	280.78	1270.00	2682.00	1623.50	1713.50	1885.00	0.2417
**Body height [cm]**	166.80	8.28	152.00	184.00	161.00	164.50	173.00	167.63	7.88	154.00	186.00	162.00	165.50	172.25	0.1485
**Body weight [kg]**	55.22	8.47	42.70	79.70	49.40	53.45	59.38	57.56	8.38	45.20	82.70	51.33	55.75	62.28	< 0.0001*
**BMI [kg/m2]**	19.78	2.03	17.00	24.90	18.70	19.15	20.70	20.41	2.02	17.40	25.50	18.70	20.10	21.50	< 0.0001*
**FM [%]**	20.70	5.86	12.90	34.50	14.88	21.45	24.48	21.72	5.67	13.90	33.90	15.78	22.30	26.05	0.0461*
**FFM [kg]**	43.79	7.52	34.30	61.20	38.45	40.50	50.58	45.07	7.58	35.50	63.50	39.38	42.45	49.75	< 0.0001*
**TBW [kg]**	32.06	5.51	25.10	44.80	28.13	29.65	37.03	32.99	5.55	26.00	46.50	28.80	31.05	36.40	< 0.0001*
**HC [cm]**	87.33	5.89	75.00	97.00	82.75	88.00	92.00	90.10	6.21	78.00	108.00	85.50	90.00	94.00	0.0001 *
**WC [cm]**	66.83	3.79	61.00	74.00	64.00	66.00	70.00	67.62	5.88	58.00	84.00	63.00	68.00	71.50	0.4632
**SBP [mmHg]**	112.03	13.63	91.00	139.00	101.00	110.00	121.75	117.38	9.80	100.00	146.00	112.00	116.00	123.50	0.0412 *
**DBP [mmHg]**	66.33	5.84	55.00	78.00	61.75	66.50	70.00	66.48	6.67	55.00	82.00	62.50	66.00	70.50	0.9571

*REE* resting energy expenditure, *FM* fat mass, *FFM* fat free mass, *HC* hip circumference, *WC* waist circumference, *TBW* total body water, *BMI* body mass index, *SBP* systolic blood pressure, *DBP* diastolic blood pressure, *Me* median, *SD* standard deviation

^*^indicate significant values (*p* < 0.05)

**Table 3:** Between-group differences from T0 to T1 by age and sex

Variables	T0							T1							***p***
	Average		Min.	Max	Q1	Q2 (Me)	Q3	Average	SD	Min.	Max	Q1	Q2 (Me)	Q3	
**Age = 9–12 years Girls (*N*** **= 1)**															
**REE [kcal]**	1221.00		1221.00	1221.00	1221.00	1221.00	1221.00	1381.00	1381.00	1381.00	1381.00	1381.00	1381.00	1381.00	
**Body height [cm]**	127.00		127.00	127.00	127.00	127.00	127.00	129.00	129.00	129.00	129.00	129.00	129.00	129.00	
**Body weight [kg]**	24.60		24.60	24.60	24.60	24.60	24.60	25.30	25.30	25.30	25.30	25.30	25.30	25.30	
**BMI [kg/m2]**	15.30		15.30	15.30	15.30	15.30	15.30	15.20	15.20	15.20	15.20	15.20	15.20	15.20	
**FM [%]**	20.30		20.30	20.30	20.30	20.30	20.30	19.70	19.70	19.70	19.70	19.70	19.70	19.70	
**FFM [kg]**	19.60		19.60	19.60	19.60	19.60	19.60	20.30	20.30	20.30	20.30	20.30	20.30	20.30	
**TBW [kg]**	14.30		14.30	14.30	14.30	14.30	14.30	14.90	14.90	14.90	14.90	14.90	14.90	14.90	
**HC [cm]**	64.00		64.00	64.00	64.00	64.00	64.00	69.00	69.00	69.00	69.00	69.00	69.00	69.00	
**WC [cm]**	54.00		54.00	54.00	54.00	54.00	54.00	57.00	57.00	57.00	57.00	57.00	57.00	57.00	
**SBP [mmHg]**	111.00		111.00	111.00	111.00	111.00	111.00	105.00	105.00	105.00	105.00	105.00	105.00	105.00	
**DBP [mmHg]**	69.00		69.00	69.00	69.00	69.00	69.00	58.00	58.00	58.00	58.00	58.00	58.00	58.00	
**Age = 13–17 years Girls (*N*** **= 16)**															
**REE [kcal]**	1533.38	210.35	1219.00	1858.00	1316.75	1588.00	1670.75	1629.75	170.81	1270.00	1985.00	1507.00	1666.00	1717.75	0.0548
**Body height [cm]**	164.81	6.65	154.00	181.00	161.25	163.00	168.00	164.69	5.38	154.00	177.00	162.00	165.00	166.75	0.8969
**Body weight [kg]**	53.29	4.89	44.10	63.40	50.58	52.30	56.48	55.38	5.11	47.30	66.20	51.58	54.55	57.70	0.0001*
**BMI [kg/m2]**	19.64	1.70	17.00	23.90	18.50	19.25	20.63	20.41	1.79	17.60	24.30	18.78	20.25	21.38	0.0246*
**FM [%]**	24.73	4.70	12.90	34.50	22.80	24.30	28.20	26.22	3.35	21.80	33.90	22.93	26.00	28.70	0.1065
**FFM [kg]**	40.09	4.28	34.30	51.20	37.23	39.30	42.63	40.80	3.61	35.50	47.50	38.40	39.70	44.03	0.0875
**TBW [kg]**	29.35	3.14	25.10	37.50	27.23	28.80	31.18	29.86	2.65	26.00	34.80	28.13	29.05	32.23	0.0916
**HC [cm]**	89.06	4.73	80.00	97.00	85.25	89.00	92.00	90.67	4.81	81.00	100.00	88.00	90.00	95.00	0.0215*
**WC [cm]**	66.50	3.85	61.00	74.00	63.25	65.50	69.75	66.27	4.04	61.00	73.00	63.00	64.00	69.00	0.5191
**SBP [mmHg]**	108.19	11.87	91.00	139.00	99.25	107.50	112.75	116.80	7.66	102.00	131.00	113.00	115.00	123.00	0.0370*
**DBP [mmHg]**	66.50	4.89	59.00	77.00	62.50	65.50	69.00	68.60	7.14	56.00	82.00	63.00	67.00	74.00	0.2390
**Age = 9–12 years Boys (*N*** **= 49)**															
**REE [kcal]**	1524.94	190.33	1187.00	2054.00	1369.50	1501.00	1631.50	1539.73	188.43	1129.00	2083.00	1419.00	1537.00	1631.50	0.4970
**Body height [cm]**	146.22	8.11	133.00	169.00	139.00	145.00	153.50	148.61	8.20	135.00	170.00	142.00	146.00	155.00	0.0000*
**Body weight [kg]**	37.77	7.44	24.80	56.90	31.85	36.20	42.10	39.60	7.67	27.20	59.50	33.10	37.80	45.40	0.0000*
**BMI [kg/m2]**	17.47	2.22	13.60	23.80	16.05	17.00	18.70	17.78	2.10	14.50	23.70	16.40	17.40	18.95	0.0000*
**FM [%]**	19.24	3.89	13.60	33.40	16.75	18.50	20.55	19.30	3.50	13.50	32.70	16.90	18.50	20.90	0.7155
**FFM [kg]**	30.33	5.08	20.60	42.30	25.90	30.00	34.00	31.81	5.38	22.30	45.10	26.65	31.40	35.75	0.0000*
**TBW [kg]**	22.21	3.72	15.10	31.00	18.95	22.00	24.90	23.29	3.94	16.30	33.00	19.50	23.00	26.20	0.0000*
**HC [cm]**	73.37	5.80	64.00	86.00	70.00	72.00	78.00	76.12	6.35	66.00	94.00	72.00	75.00	79.00	0.0001
**WC [cm]**	58.31	7.49	21.00	73.00	55.00	58.00	61.50	60.18	9.84	6.00	81.00	57.00	59.00	63.50	0.2280
**SBP [mmHg]**	105.51	12.44	85.00	141.00	95.50	104.00	114.00	105.57	11.02	87.00	132.00	98.00	103.00	112.00	0.9660
**DBP [mmHg]**	60.29	6.44	47.00	74.00	56.00	59.00	65.00	61.53	6.68	50.00	80.00	56.00	62.00	66.00	0.2951
**Age = 13–17 years old Boys (*N*** **= 14)**															
**REE [kcal]**	1948.43	315.75	1582.00	2639.00	1717.25	1843.50	2240.00	1937.57	296.29	1606.00	2682.00	1744.50	1869.00	2179.50	0.8645
**Body height [cm]**	169.07	9.57	152.00	184.00	160.25	170.00	177.25	171.00	9.06	156.00	186.00	163.25	171.50	179.25	0.0002*
**Body weight [kg]**	57.42	11.08	42.70	79.70	47.50	56.80	65.70	60.05	10.68	45.20	82.70	50.85	59.55	68.35	0.0000*
**BMI [kg/m2]**	19.94	2.40	17.10	24.90	18.50	19.05	21.25	20.41	2.32	17.40	25.50	18.70	19.60	21.85	0.0006*
**FM [%]**	16.11	2.89	13.40	23.20	13.75	15.20	18.40	16.59	2.35	13.90	23.20	15.15	15.75	17.55	0.1653
**FFM [kg]**	48.01	8.31	35.70	61.20	40.00	48.45	55.23	49.96	8.06	38.30	63.50	42.68	50.00	57.25	0.0000*
**TBW [kg]**	35.15	6.08	26.10	44.80	29.33	35.45	40.40	36.56	5.91	28.00	46.50	31.23	36.60	41.93	0.0000*
**HC [cm]**	85.36	6.62	75.00	97.00	80.75	84.00	89.50	89.50	7.57	78.00	108.00	84.00	88.00	93.75	0.0018*
**WC [cm]**	67.21	3.83	61.00	74.00	65.00	66.00	71.00	69.07	7.25	58.00	84.00	63.50	69.50	75.25	0.3548
**SBP [mmHg]**	116.43	14.59	96.00	138.00	101.00	113.00	130.00	118.00	11.96	100.00	146.00	109.50	119.00	126.25	0.5853
**DBP [mmHg]**	66.14	6.97	55.00	78.00	59.75	67.50	71.25	64.21	5.49	55.00	74.00	60.75	64.50	68.50	0.3376

*REE* resting energy expenditure, *FM* fat mass, *FFM* fat free mass, *HC* hip circumference, *WC* waist circumference, *TBW* total body water, *BMI* body mass index, *SBP* systolic blood pressure, *DBP* diastolic blood pressure, *Me* median, *SD* standard deviation

^*^indicate significant values (*p* < 0.05)

We noticed that REE was not significantly different between baseline (1596.94 ± 273.01 kcal) and after 10 months (1625.38 ± 253.26 kcal). When divided into two groups: children (9–12 years) and adolescents (13–17 years), in both groups there were no significant differences in REE values. Furthermore, both in girls and boys, there were no significant differences in REE.

A significant difference in body weight was observed in younger children (37.51 ± 7.60 kg vs 39.31 ± 7.86 kg), and older children (55.22 ± 8.47 kg vs 57.56 ± 8.38 kg) children, but body height was significantly higher only in younger children (145.84 ± 8.48 cm vs 148.22 ± 8.57 cm). Additionally, both at baseline and after 10 months, the increase in fat free mass was observed (*p* < 0.05). However, fat mass (%) increases significantly only in adolescents (20.70 ± 5.86% vs. 21.72% ± 5.67%, *p* = 0.0461).

Significant differences in hip circumference were observed between the two measurement points (*p* = 0.0009). The value has increased in both younger and older children (*p* < 0.05).

In adolescents aged 13–17 years, an increase in systolic blood pressure was observed from 112.03 ± 13.63 mmHg to 117.38 ± 9.80 mmHg (*p* = 0.0412).

In Table 3 the differences between the sexes and age are presented. We found significant differences in body weight, BMI, hip circumference, and systolic blood pressure in girls (*p* < 0.05). In boys, significant differences were the same in the younger (9–12 years) and older (13–17 years) groups, and differences have been observed in body height, body weight, BMI, FFM, total body water, and hip circumference.

The differences between girls and boys are presented in Table 4.

**Table 4:** The differences in variables between girls and boys in T0 and T1

Sex	Girls		Boys		Total		*p*
	Average	SD	Average	SD	Average	SD	
**Differences between T0 and T1**							
REE	100.12	179.87	9.10	171.05	28.44	175.84	0.0577
Age [years]	0.41	0.51	0.30	0.46	0.33	0.47	0.3958
Body height [cm]	0.00	3.71	2.29	1.11	1.80	2.15	0.0001*
Body weight [kg]	2.01	1.54	2.01	1.00	2.01	1.13	0.9988
BMI [kg/m2]	0.72	1.22	0.34	0.46	0.42	0.70	0.0470*
FM [%]	1.37	3.41	0.16	1.21	0.41	1.94	0.0208*
FFM [kg]	0.71	1.50	1.58	0.80	1.40	1.04	0.0017*
TBW [kg]	0.52	1.10	1.16	0.58	1.02	0.76	0.0017*
HC [cm]	1.75	2.38	3.06	4.33	2.80	4.03	0.2472
WC [cm]	−0.13	2.06	1.87	10.03	1.47	9.03	0.4327
SBP [mmHg]	7.19	13.56	0.40	10.05	1.77	11.10	0.0279*
DBP [mmHg]	1.13	6.71	0.54	8.08	0.66	7.78	0.7902

*REE* resting energy expenditure, *FM* fat mass, *FFM* fat free mass, *HC* hip circumference, *WC* waist circumference, *TBW* total body water, *BMI* body mass index, *SBP* systolic blood pressure, *DBP* diastolic blood pressure, *SD* standard deviation

^*^indicate significant values (*p* < 0.05)

Due to the large number of statistically significant differences between girls and boys, stepwise linear regression was performed separately for both sexes (Table 5).

**Table 5:** The result of the general regression model for the selected parameters (independent variables were selected by the stepwise forward regression procedure)

Model		Non-Standardized Coefficients		Standardized Coefficients	t	***p***
		B	SE (B)	β		
Girls (T0)	FFM [kg]	21.825	4.656	0.650	4.687	0.0003*
	DBP [mmHg]	26.158	6.321	0.574	4.138	0.0010*
Boys (T0)	TBW [kg]	46.443	6.131	1.133	7.575	0.0000*
	Age [years]	−42.168	20.492	−0.308	−2.058	0.0440*
Girls (T1)	Body height [cm]	8.692	4.036	0.499	2.153	0.0492*
Boys (T1)	TBW [kg]	32.110	2.978	0.839	10.782	0.0000*
	DBP [mmHg]	−8.339	3.254	−0.199	−2.563	0.0129*
Girls (difference between T0 and T1)	Body height [cm] - difference	34.147	9.252	0.702	3.691	0.0024*
Boys (difference between T0 and T1)	No significant predictors for REE					

*REE* resting energy expenditure, *FFM* fat free mass, *TBW* total body water, *DBP* diastolic blood pressure, *SE* standard error

B—regression coefficient, p—significance of regression coefficient, β—standardized regression, coefficient, t—Student t Test

^*^indicates significant values (*p* < 0.05)

We noticed that at baseline, REE was influenced by FFM and diastolic blood pressure in girls (higher FFM and pressure = higher REE). In the baseline group of boys, REE was influenced by TBW (higher TBW, higher REE) and age (higher age, lower REE).

After 10 months, REE was influenced by body height in girls (higher height means higher REE, β = 0.499). In the group of boys, the REE was influenced by TBW (positive, that is, higher TBW, higher REE results) and diastolic pressure (but it was negative, that is, higher diastolic pressure, lower REE (β = − 0.199).

When analyzing the difference in REE between the studies in girls, we only had one significant predictor, which turned out to be the difference in body height. The greater the difference in body height, the greater the difference in REE between T0 and T1. In the group of boys, there was no significant predictor that would influence the change in REE (i.e., the difference in REE between T0 and T1).

## Discussion

This is the first longitudinal analysis to examine changes in REE and body composition in healthy children and adolescents who play sports regularly in Poland. This is a very important issue, as changes in body and energy expenditure with growth and age are relevant in the population of young football players. Longitudinal studies of REE are rare, particularly in children and adolescents, due to the high costs associated with repeated examinations of REE. The main purpose of our study was to check if with the age and increase of FFM (the greatest predictor of REE change), body height and body weight gain, the REE value will also change.

### Resting energy expenditure

Data show that body weight gain before puberty is associated with an increase in REE and that the increase is greater than predicted from changes in body composition [33]. Our results showed that age was not related to the measured REE in the total sample. Furthermore, when the study group was divided into two sub-groups: children (9–12 years) and adolescents (13–17 years), the REE also did not increase significantly from baseline. This is consistent with existing evidence [34] and is true for children in middle school through age and sex categories and the population groups we examined. Therefore, we have received support for our hypothesis that body weight gain and changes in body composition in children and adolescents elicit adaptive changes in REE.

### Body composition

Furthermore, there was no association between body mass gain or change in body composition and REE in girls and boys. Therefore, our hypothesis that REE has a significant impact on changes in body weight or composition is refuted. The results of the literature in both adults [35, 36] and children [37–39] have been inconsistent, mainly due to different age ranges and populations. In the study by Broadney et al., the lower REE in African American children was likely due to a lower trunk lean mass and a greater appendicular lean mass. In addition, they noticed that differences in the distribution of lean mass may largely explain the observed lower REE in African-American children compared to Caucasian-American children [15]. However, it was not a longitudinal study. Sun et al. took a sample of children and prospectively monitored body composition and REE throughout puberty. Unlike the Broadney study, they did not identify attenuation in REE racial differences by adjusting for compartment-specific lean mass [40].

In the study by Hosking et al. relative to changes in body composition, there were little or no significant changes in REE prior to age 9 to 10 years [41]. There were only a few longitudinal studies with changes in REE in children [19, 36, 39, 42]. In different studies, the TEE adjusted for FFM did not differ significantly from 10.4 to 12.8 years [36]. In addition, Spadano et al., similar to our study, noticed that the mean REE in children 12 years of age did not differ significantly from the REE in 15 years of age [42]. However, we found significant increases during growth in both FFM, the most important determinant of REE [43] and FM [44, 45] in older children, an independent contributor to REE. These mentioned studies have shown a dependence on age and body composition.

### Main predictors

When analyzing the results with stepwise linear regression, we found predictors of REE in boys and girls. Body height turned out to be a predictor of the change in the REE difference between studies. However, this result was only significant in girls. The greater the difference in body height, the greater the difference in REE between baseline and after 10 months. A taller person differs from a shorter person of the same body weight in their relative amounts of adipose tissue, muscle, and other organs and tissues. Therefore, body mass alone is an inadequate phenotypic marker of the body size of an adult human and therefore its REE. People who are tall, given the same age and level of fat (%) will weigh more than people who are short [46].

### Limitations

The study has some limitations. Limitations of resources required the use of BIA instead of a gold standard measure, such as dual energy X-ray absorptiometry (DXA) for analysis of body composition, and a portable device (Fitmate Med). This may lead to errors compared to the gold standard. Despite not measuring CO_2_ production, it is very convenient in the clinical setting to assume a minimal analysis error. In addition, environmental factors, such as food intake and physical activity, could help regulate the overall regulation of energy balance. Young soccer players are grouped by chronological age to reduce the effects of developmental differences. However, young athletes of the same chronological age can vary in their maturity status (stage of puberty, skeletal age, maturity timing) therefore another limitation of our study was not measuring the Tanner stages to directly indicate the stage of puberty, which may influence on the results. We had got large age ranges, numerically small files, and did not take into account maturation. Finally, there were many environmental and epigenetic influences that could affect the results (health status, children’s morbidity, injuries, and nutrition).

## Conclusions

In conclusion, the results of our study show a negative relationship between growth, age, and REE. The differences between sexes and age in REE between baseline and 10 months after were not significant. Although lean body mass appears to be the largest predictor of REE in physically active people, in our study, despite a significant increase in FFM, REE did not increase significantly. To summarize, a study involving physically active children and adolescents, which used repeated objective measures and longitudinal statistical modeling to analyze them, was unable to demonstrate any interaction between body weight change, body composition measurements, and REE after 10 months. In Poland, actual REE measurement is not feasible in most clinical and research settings. The importance of REE lies in its potential to influence weight gain, and although the role of REE in future body weight change remains controversial, we have been unable to support the hypothesis that it increases with chronological age.

## Supplementary Information

**Additional file 1.**

## Data Availability

The dataset supporting the conclusions of this article is included within the article (and its additional file).

## Abbreviations

BMI: Body mass index
EFSA: European Food Safety Authority
FAO: Food and Agriculture Organization of the United Nations
FFM: fat-free mass
NHBPEP: National High Blood Pressure Education Program Working Group in Children and Adolescents
PA: Physical activity
REE: Resting energy expenditure
TBW: Total body water
TEE: Total energy expenditure
UNU: United Nations University
WHO: World Health Organization
